# Single-Cell Transcriptome and RNA Sequencing Reveal Immune-Related Markers of Preeclampsia

**DOI:** 10.1007/s43032-025-01843-5

**Published:** 2025-04-11

**Authors:** Xiaoting Yu, Huiqiu Xiang, Xianping Huang

**Affiliations:** Department of Gynecology and Obstetrics, The Second Affiliated Hospital of Wenzhou Medical University, No.306 Hualongqiao Road, Lucheng District, Wenzhou City, Zhejiang Province 325027 The People’s Republic of China

**Keywords:** Preeclampsia, Immune microenvironment, Macrophages, Single-cell transcriptome, RNA-sequencing

## Abstract

Preeclampsia is a leading cause of maternal and fetal mortality, posing a threat to the mother and fetus’s lives, but its pathogenesis is not yet clear. This study aimed to find the key target genes regulating preeclampsia in the placental immune microenvironment through single-cell and RNA-sequencing. This study compared the immune microenvironment of the placenta in preeclamptic and non-preeclamptic samples. Gene Ontology Enrichment (GO), Kyoto Encyclopedia of Genes and Genomes (KEGG), pseudotime, and cell-cell communication techniques were utilized to analyze the association between immune cell function and the pathogenesis of preeclampsia. Specific transcription factor target genes of immune cells were obtained based on the Scenic package and intersections were taken with their differentially expressed genes (DEGs). Key differential genes analysis was performed on the intersected genes based on the *GSE234729* and *GSE25906* datasets to obtain differential target genes regulating preeclampsia in immune cells. A total of 10 cell types were annotated in the placenta. Among them, macrophages had the highest immune score, followed by monocytes. GO and KEGG results demonstrated that they might be involved in inflammatory response and vascular remodeling in preeclampsia. Macrophages in the placenta were reclustered and annotated to monocytes, macrophages, and Hofbauer cell subsets, of these, monocytes could differentiate into macrophages and Hofbauer cells. Of all the immune cell-regulated preeclampsia differential genes SLC9A9, SH2B3, SDC3, RCC2, F13A1, CCL2, and CBLB were consistently expressed in two transcriptome datasets, and all were highly expressed in macrophages. These findings suggested that macrophages were implicated in the aberrant immune and inflammatory response of the preeclamptic placenta and found its key target genes that regulate preeclampsia.

## Introduction

Preeclampsia is a complication that occurs only during pregnancy, affecting 4-5% of maternal pregnancies worldwide and killing 76,000 women and 500,000 babies each year [1–3]. Preeclampsia is a complex condition caused by various factors, processes, and pathways [4]. The main symptoms include sudden onset of high blood pressure and proteinuria, and in severe cases, even liver and kidney damage, along with the neurological symptoms of dizziness, nausea, and vomiting [5–7]. If left untreated, maternal generalized tension syndrome, fetal growth retardation, preterm delivery, and stillbirth may occur [8]. Preeclampsia poses a serious risk to the life and health of mother and fetus, therefore, an in-depth comprehension of its pathogenesis is extremely important.

Normal pregnancy is considered a state of “immune tolerance” as the maternal immune system needs to function properly without attacking the fetus. In a normal pregnancy, there is a complex interaction between the mother’s immune system and placenta that allows the fetus to grow in the womb. The placenta is the organ that nourishes the fetus throughout pregnancy, and in early pregnancy, new blood vessels develop and evolve to provide oxygen and nutrients to the placenta. In women with preeclampsia, these blood vessels do not seem to develop or work properly. Problems with placental circulation cause abnormal regulation of the mother’s blood pressure and the placental blood vessels failing to expand properly, bringing about restricted blood flow [9, 10]. The exact mechanism of preeclampsia is not yet fully understood, and current research suggest that the pathophysiology and mechanisms of preeclampsia are multifactorial, primarily involving failure of spiral artery remodeling and placental vascular endothelial dysfunction [11, 12]. An important feature of preeclampsia is the overactivation of the maternal inflammatory immune response [13, 14]. In normal placenta, macrophages, NK cells, and other cells in the placenta during preeclampsia act as immune cells to regulate the maternal immune balance, and these cells not only have immunomodulatory functions, but participate in the regulation of trophoblast invasion, angiogenesis, and spiral artery remodeling [15–17]. When an abnormal immune response exists in the placenta, the abnormal immune response continuously releases pro-inflammatory factors into the circulation, inducing placental vascular inflammation. At the same time, the increased release of anti-angiogenic substances induces a decrease in utero-placental blood flow, resulting in an ischemic or hypoxic environment in the placenta, further accelerating placental dysfunction and promoting the development of preeclampsia [18, 19]. Hence, studies have pointed out that the use of anti-inflammatory or pro-angiogenic drugs can control the condition [20, 21]. The pathogenesis of preeclampsia is complicated, and the current treatments can only slow down the condition effectively, so an insight into the relationship between preeclampsia and the immune response may contribute to grow new therapeutic strategies.

This study collected single-cell data from preeclampsia and non-preeclampsia patients from the GEO database for analysis, to screen the cellular subsets that play a major role and their possible mechanisms of action in regulating preeclampsia, and finally to predict differential target genes of the targeted subsets that regulate preeclampsia at the transcriptional level, which could serve as potential disease markers for the treatment of preeclampsia.

## Methods

### Data Collection and Quality Control

This study used single-cell RNA-sequencing data of 4 samples (2 preeclamptic placental samples, 2 normal samples) from the *GSE173193* dataset in the GEO database (https://www.ncbi.nlm.nih.gov/geo/*)*, and RNA sequencing data of 171 samples (66 preeclamptic placental samples, 105 normal samples) from the *GSE234729* and *GSE25906* datasets. Quality control was performed for single cells in each sample. and cells with nFeature_RNA below 200 and above 6,000, nCount_RNA below 1,000 and above 30,000, and mitochondrial content above 10% were discarded. Principal component analysis was used to dimensionalize the cell cycle-related genes and to see the effect of these genes on cell clustering.

### Single-Cell RNA-Sequencing Data Analysis

The Seurat package (versions 5.0.1) performed normalised dimensionality reduction on the raw data of GSE173193 dataset and clustering using Uniform Manifold Approximation and Projection to obtain all cell clusters, marker genes in each cluster were identified by referring to relevant literature [22], after which the cell types were annotated in conjunction with SingleR (versions 2.4.1), and filtered according to the corresponding parameters (*p* < 0.05,|log2FC|>1) to identify the differential genes in each subcluster to generate cell types.

### Identification and Scoring of Differentially Expressed Immune-related Genes

Immunity-related genes were downloaded from Immport database, and the intersection with single-cell differential genes was taken to obtain immunity-related differential genes, which were used for scoring all single cells using AUCellR (versions 1.22.0) software package. In general, cells with high expression of differential genes had higher AUC values, thus screening for immune-related cell subset.

### Analysis of Differential Gene and Functional Enrichment of Cell Subtypes

Differential genes in cells were screened according to the corresponding parameters (*p* < 0.05,|log2FC|>1). The candidate genes related to cells were analyzed for GO and KEGG functional enrichment based on the ClusterProfiler package (versions 4.8.1). Each entry in the analysis results corresponds to a statistical value p-value to indicate the significance, and a smaller p-value indicates that the entry is more linked to the input gene.

### Pseudotime Analysis

Monocle2 package (versions 2.28.0) was used to explore the developmental differentiation profile of macrophages in order to probe the impact of macrophages in preeclampsia. DDRTree (versions 0.1.5) was used to learn tree-like trajectories. The heatmap along the developmental trajectory was shown for marker genes of macrophage subtypes.

### CellChat Cell Communication Analysis

Based on CellChat package (versions 1.6.1), inter-cell communication network and internal regulatory signals are inferred by integrating intra-cellular and inter-cellular signals, and inter-cell interactions are reflected by cell-cell communication scores.

### Scenic Transcription Factor Regulatory Network

Gene regulatory networks were inferred using the Scenic package (versions 1.3.1) and based on co-expression and DNA motif analysis. The network activity was analyzed in each cell to identify the cellular state to find transcription factors and their target genes that are associated with differential function in macrophages to identify the transcriptome regulation that leads to differential gene expression and functional differences.

### Transcriptomics to Verify Differential Gene Expression

Differential analysis of key target genes data in the *GSE234729* and *GSE25906* datasets using TinyArray (versions 2.3.2) to identify key genes related to preeclampsia regulated by macrophages. Differential genes in cells were screened according to the corresponding parameters (*p* < 0.05,|log2FC|>1).

## Results

### Single-Cell Transcriptome of Preeclamptic and Control Placentas

**Fig. 1:**
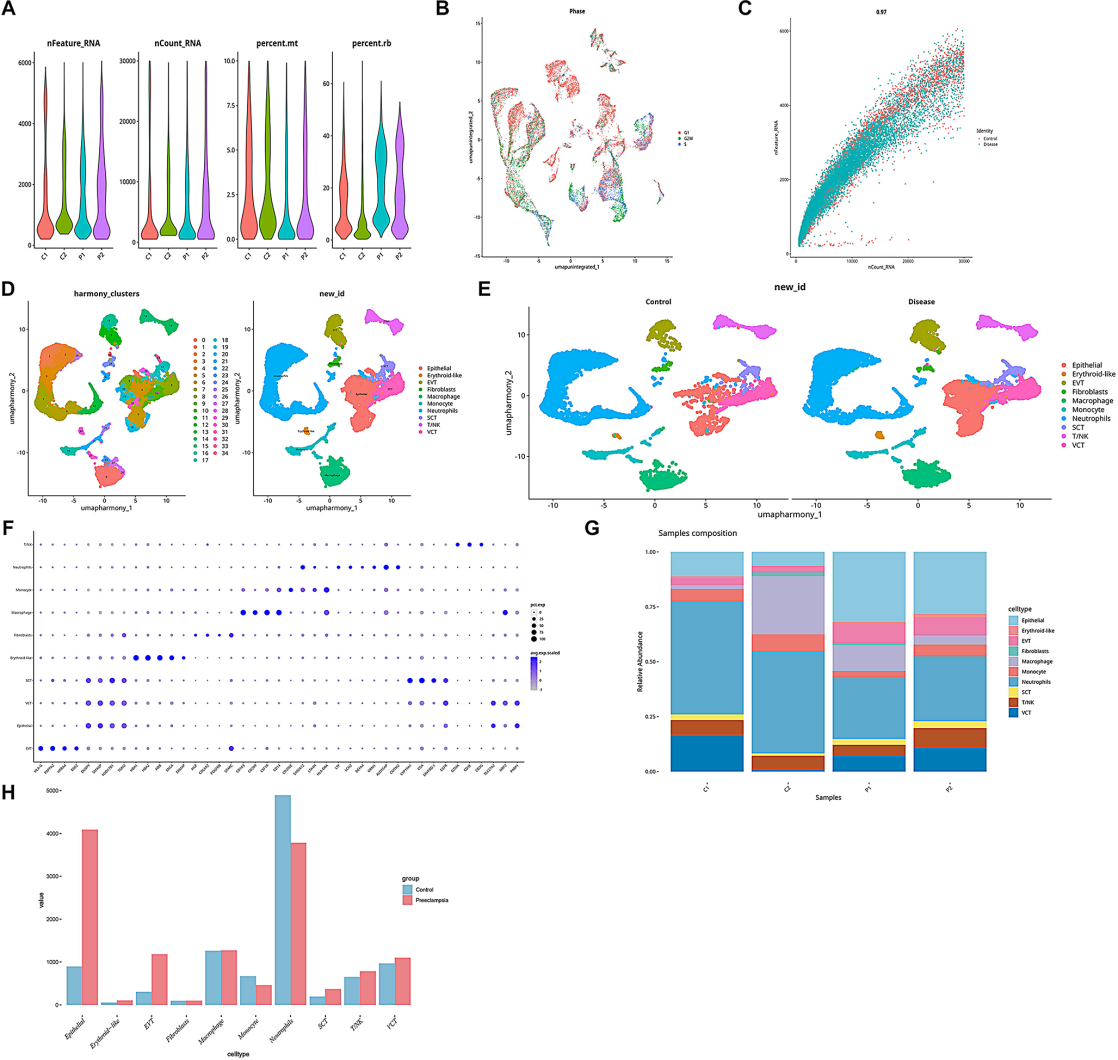
Single-cell transcriptome of preeclamptic and control placentas. (**A**) Quality control of single-celldata; (**B**) Cell cycle scores; (**C**) Correlation plots of nCount_RNA with nFeauture_RNA; (**D**) Cell clustering and cell type annotation results; (**E**) Distribution of all cell types in control and preeclampsia groups; (**F**) Bubble plots of marker gene expression corresponding to each cell type. Epithelial cells: DUSP9, SMAGP, HSD17B1; Erythroid-like cells: HBA1/2, HBB/SNCA; EVT: HLA-G, PAPPA2, HTRA4, fibroblasts: COL6A2, HGF, PDGFR8; Macrophages: CD163, CD209, CSF1R, Monocytes: CD300E, CD14, S100A12; Neutrophils: LTF, LCN2, DEFA4; SCT: CYP19A1, CGA, ERVFRP-1; T/NK cells: CD3E, CD3G; VCT: NRP2, EGFR, SCL27A2. (**G**) The percentage of cell types in each sample; (**H**) The percentage of cell types in the preeclampsia and control groups

Single-cell RNA-sequencing data from GSE173193 database including 4 samples (2 non-preeclampsia samples, n_*control*_=2, and 2 preeclampsia samples, n_*preeclampsia*_=2) were analyzed, and low-quality or dead cells were removed after quality control (Fig. 1A). Cell cycle assessment revealed that the cell cycle (G1, G2/M, S) had no effect on clustering (Fig. 1B), and both the gene expression levels and the number of genes showed high correlation between different group (Fig. 1C). UMAP visualized and clustered all the cells, 35 cell clusters were obtained. By characterizing marker genes, they were annotated to 10 major cell types, namely epithelial cells, erythroid-like cells, chorionic extravillous trophoblast cells (EVT), fibroblasts, macrophages, monocytes, neutrophils, syncytiotrophoblast (SCT), T/NK cells, and villous cytotrophoblast cells (VCT) (Fig. 1D, F). Epithelial cells and neutrophils were higher in all cell types, and epithelial cells were much higher than the control in the preeclampsia group, whereas monocytes were significantly more numerous in the control group, and there was no clear pattern of change in the other cell types (Fig. 1E, G-H).

### Immunoscoring Results for Single Cells

**Fig. 2:**
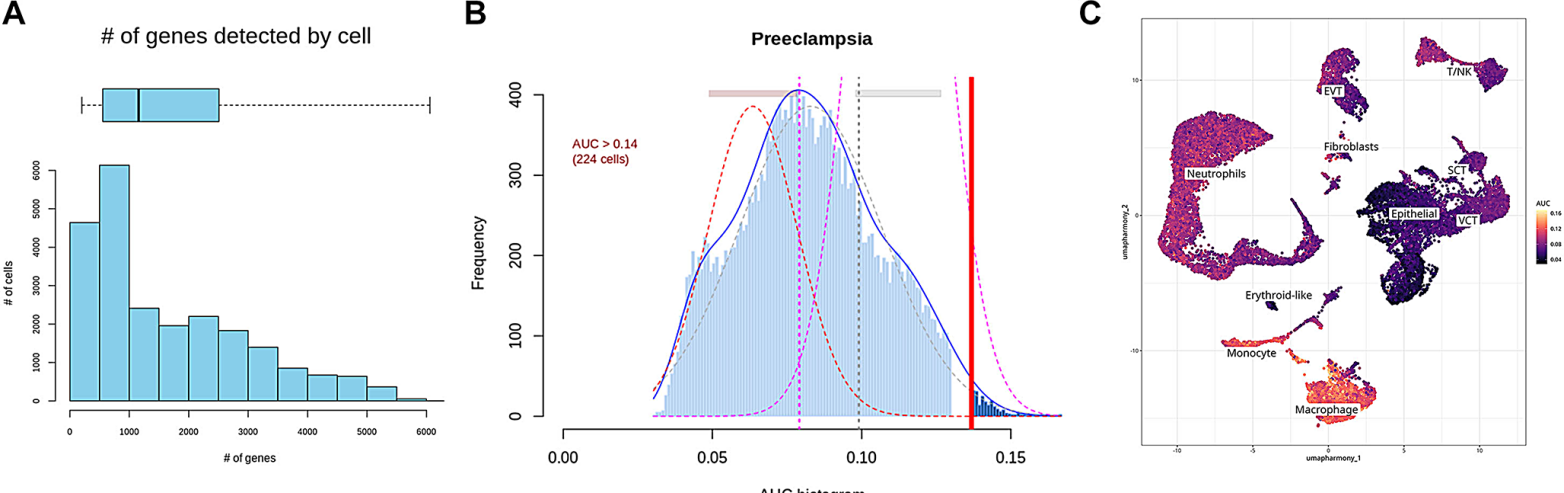
Immunoscoring results for single cells. (**A**) The number of immune genes contained in each cell; (**B**) Distribution of immune-related AUC scores in each cell; (**C**) The immune-related scores of all cell types

Since study have been pointed out that the development of preeclampsia may be related to abnormal immune regulation, so we scored the expression of immune-related genes in all cell types. As shown in Fig. 2A and B, we obtained the distribution of the number of immune genes contained in each cell and the immune-related AUC score, in which 250 cells were more relevant to immunity. The scoring results were subsequently presented in a heatmap, which revealed that the highest immune score was macrophages, followed closely by monocytes, and the lowest was epithelial cells (Fig. 2C). Therefore, the role of macrophages and monocytes in preeclampsia will be analyzed further.

### Preeclampsia Is Associated with Immune and Inflammatory Response in Placenta

**Fig. 3:**
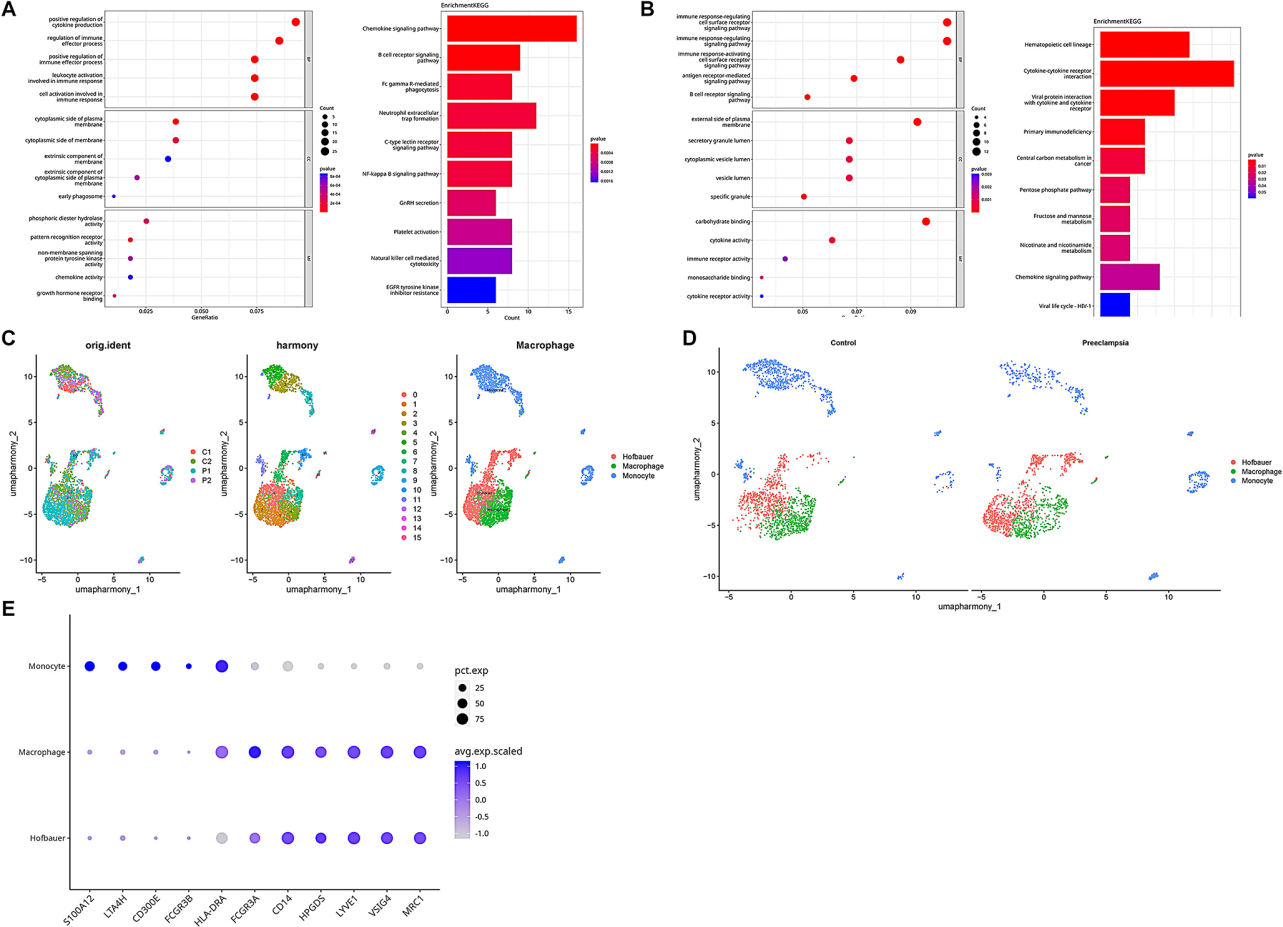
Preeclampsia is associated with immune and inflammatory response in placenta. The GO and KEGG enrichment result of DEGs in (**A**) macrophages and (**B**) monocytes; (**C**) Macrophages re-clustering and annotation, with the distribution of cells in the sample on the left, all cell clusters in the middle, and subtype annotation results on the right; (**D**) Distribution of macrophages subtypes in the control and preeclampsia groups; (**E**) Bubble plots of marker genes expression corresponding to the three subtypes (Macrophages: HLA-DRA, FCGR3A; Monocytes: S100A12, CD300E; Hofbauer cells: HPGDS, VSIG4)

Analysis of differentially expressed genes (DEGs) and functional enrichment of DEGs were performed for macrophages and monocytes between the control and preeclampsia group. The GO enrichment results revealed that compared with the control group, DEGs of macrophages in the preeclampsia group were enriched in “positive regulation of cytokine production”, “regulation of immune effector processes”, “cell activation involved in immune response”. Similarly, DEGs of monocytes in the preeclampsia group were enriched in the “immune response-regulating cell surface receptor signaling pathway” and “immune response-regulating signaling pathway”. “Chemokine signaling pathway” was enriched in both macrophages and monocytes in KEGG enrichment results (Fig. 3A-B). Therefore, macrophages and monocytes may be participated in the over-activation of immune and inflammatory responses in preeclampsia placenta. We then redefined all the macrophages in the placenta and obtained 16 macrophages subclusters, which could be subdivided into macrophages, monocytes, and the placenta-unique Hofbauer cells after cell annotation [23, 24] (Fig. 3C, E). In the control group, there were more monocytes and Hofbauer cells, whereas macrophages were more prevalent in the preeclamptic group, which suggested that macrophages play an important role in preeclampsia (Fig. 3D).

### Differentiation of Macrophages in the Preeclamptic Placenta

**Fig. 4:**
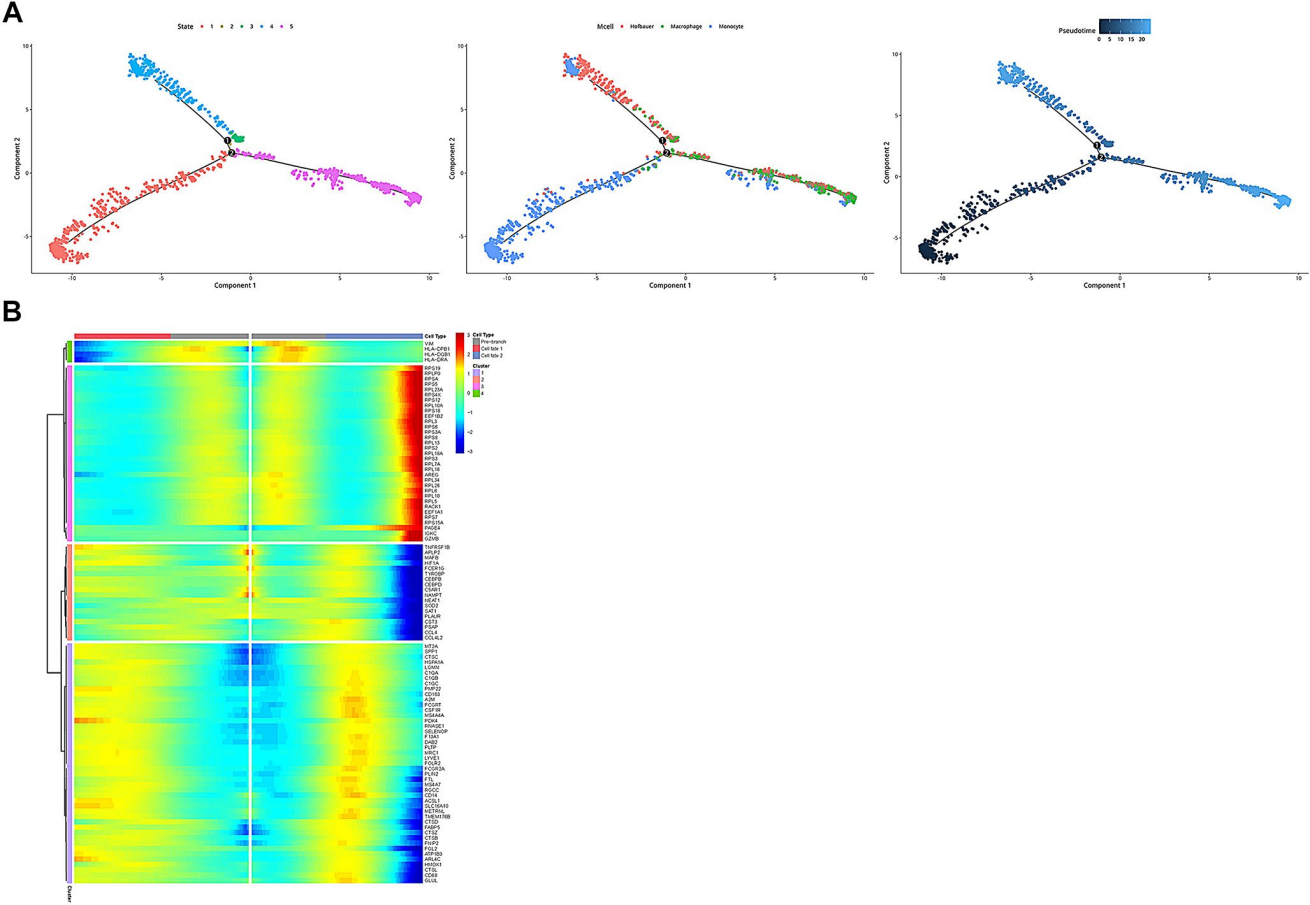
Differentiation of macrophages in the preeclamptic placenta. (**A**) Differentiation trajectories of macrophages, monocytes, and Hofbauer cells. The left: simulated time phase, each color represented a different phase; The middle: distribution of different subtypes in the pseudotime; The right: cell differentiation trajectory, the darker the color the earlier the period; (**B**) Cluster time simulations were performed according to differentiation scenarios and plotted as heatmaps

With the aim to explore which macrophage subtype plays a crucial role in preeclampsia, pseudotime was used to analyze the differentiation of macrophage subtypes, and found that monocytes could differentiated into macrophages and Hofbauer cells (Fig. 4A). By modelling the expression of genes in each of the three subtypes, it was found that the expression levels of these genes changed as the subtypes diverged (Fig. 4B). HLA-DRA, HLA-DPB1, and HLA-DQB1 were highly expressed in the monocytes but reduced in the differentiated macrophages and Hofbauer cells, they were expressed on the surface or inside antigen-presenting cells and played central roles in the immune system. We also tagged the gene expressions related to immune and inflammatory responses. The immune receptor activation signal transduction, such as CEBPB, FCER1G, and CCL4 decreased in their levels following cell differentiation. Besides, genes such as APLP2 and HIF1A that played a role in the regulation of vascular endothelial growth factor functions decreased expressions. In summary, macrophage subtypes were related to the aberrant immune regulation in preeclampsia, in which monocytes mediated the imbalance of immune and inflammatory responses and placental angiogenesis through differentiation into macrophages and Hofbauer cells.

### Interaction of Macrophages with Other Cell Types in Preeclamptic Placenta

**Fig. 5:**
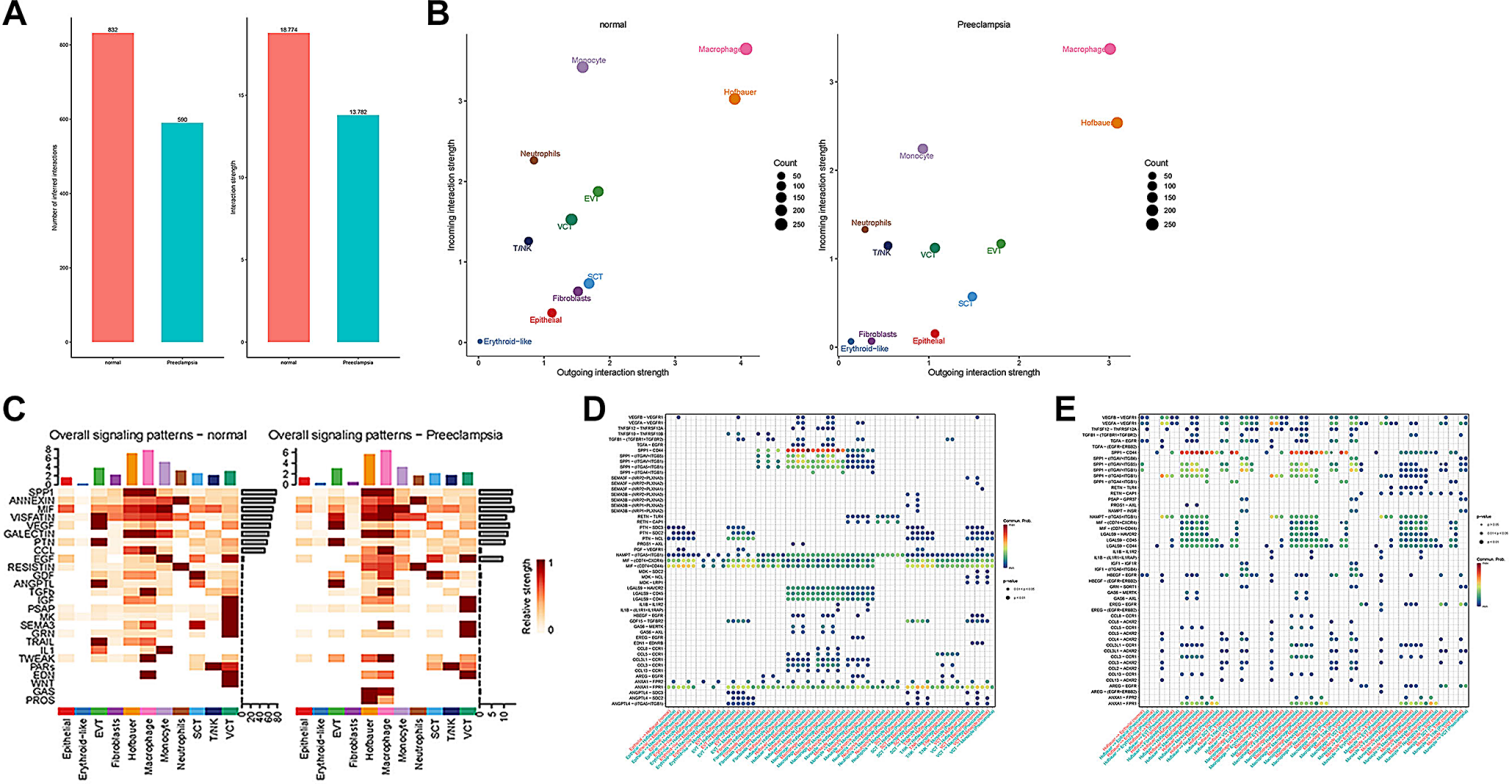
Interaction of macrophages with other cell types in preeclamptic placenta. (**A**) Statistical plots of the total number of communications and strength of communications for all cell types in the control and preeclampsia groups; (**B**) Two-dimensional projection plots of efferent and afferent signals of different cell types; (**C**) The strength of the different signaling pathways in each cell type; (**D**) Other cells influenced the receptor-ligand pairs of three macrophage subpopulations; (**E**) Three types of macrophage subpopulations influenced the receptor-ligand pairs of other cells

To further discover the function of macrophages in preeclampsia, we analyzed the intercellular communication between cell types in the control and preeclampsia groups. Results suggested that the number of communications between cell types in the control group was more numerous and stronger (Fig. 5A-B). These cell types mainly functioned in SPP1, Annexin, MIF, Visfatin, VEGF, Galectin, PTN, and CCL signalings. Compared with the control group, the preeclampsia group showed a decrease in communication in different signaling pathways, with a higher decrease in CCL and Visfatin signalings (Fig. 5C), indicating that preeclampsia inhibited normal communication between placental cells. Additionally, communication between the three subtypes of macrophages frequently, which implied that they play a very critical role in the overall function (Fig. 5D-E). So, we assessed the receptor-ligand pairs that associated with these three cell subtypes, and the SPP1-CD44 pairs had the strongest roles in communication, and thus may be a potential point of action for the progression preeclampsia.

### Macrophages as a Major Cell Type to Regulated Preeclampsia

**Fig. 6:**
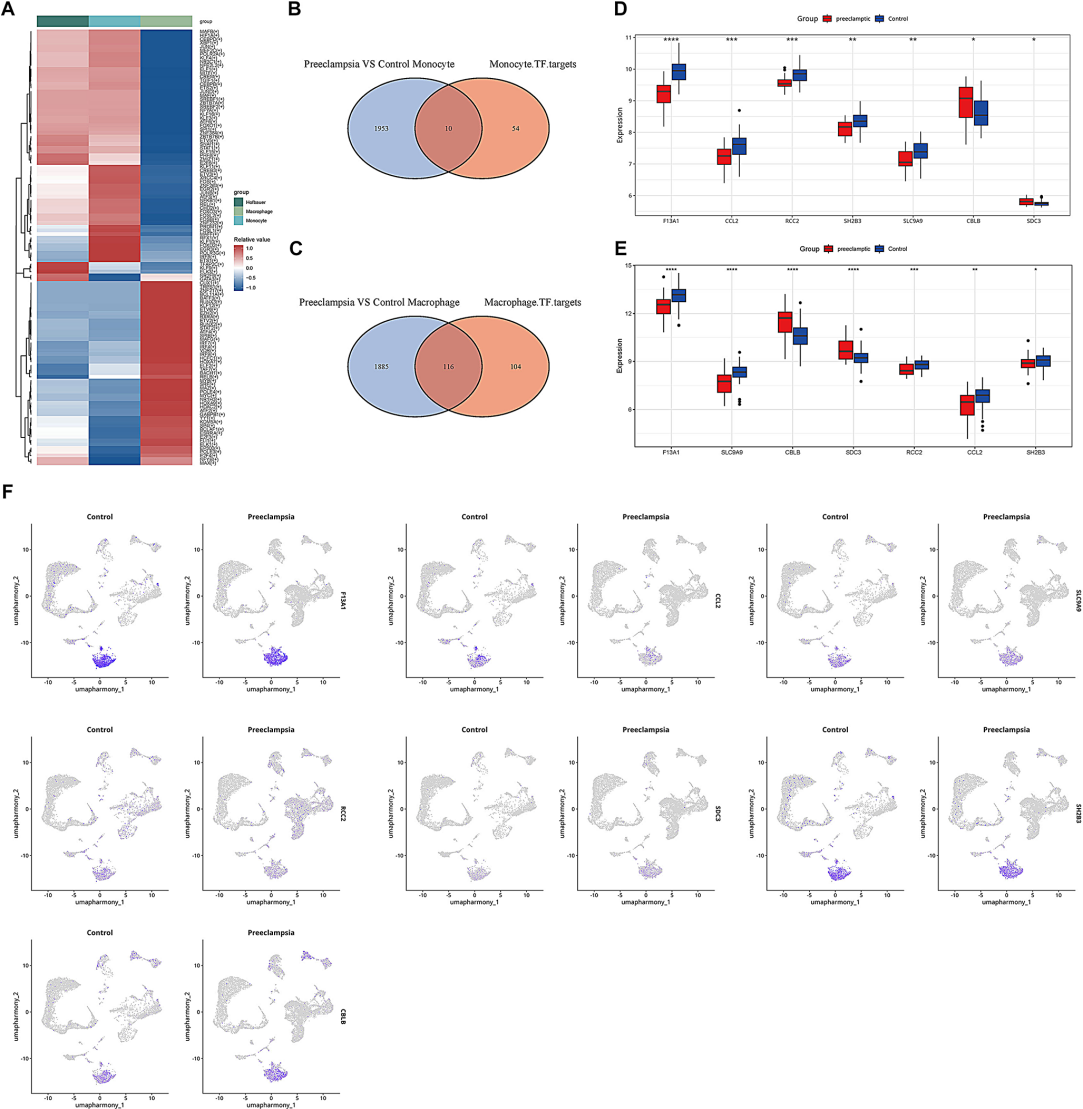
Macrophages as a major cell population to regulated preeclampsia. (**A**) Heatmap display of differential transcription factors regulating macrophage, monocyte, and Hofbauer cells; (**B**) Transcription factor target genes of monocytes and (**C**) macrophages were intersected with DEGs; (**D**) Analysis of key differential genes in the GSE25906 and (**E**) GSE234729 datasets; (**F**) Differential expression of screened key differential genes in different grouped cell types

In order to clarify the mechanism of macrophages in regulating preeclampsia, we analyzed the transcription factors target genes of the three subtypes in the preeclampsia group (Fig. 6A). In the previous analysis, we got the DEGs of macrophages and monocytes between the control and the preeclampsia group. Then intersected the DEGs and the differential transcription factor target genes, and screened out 10 differential target genes related to preeclampsia in monocytes and 116 in macrophages (Fig. 6B-C). These genes were further assessed based on the GSE234729 and GSE25906 datasets and identified 7 genes (SLC9A9, SH2B3, SDC3, RCC2, F13A1, CCL2, and CBLB) were differentially expressed with a consistent trend in the transcriptome, and that they were highly expressed in macrophages (Fig. 6D-F). Thus, macrophages participated in the regulation of preeclampsia as an important functional cell type in the placenta.

## Discussion

The process of placenta development is a complex and highly regulated process where each cell type needs to dynamically respond to the microenvironment to fulfil its ultimate fate and function [25]. It has been reported that some immune cells from the mother are recruited to uterine tissues in order to regulate vascular remodeling and angiogenesis [26]. Whereas, the abnormal working of the placenta causes a local or systemic inflammatory response. In this study, by deeply dissecting the immune microenvironment in preeclamptic placenta, 10 cell types were obtained, of which immune cells (macrophages, neutrophils, monocytes, T/NK cells) accounted for a relatively large proportion. The key to the pathogenesis of preeclampsia lies in the dysregulation of the immune system on the placenta thereby causing a series of organismal dysfunctions, therefore, this study focuses on macrophages and monocytes, which have the highest immune-related scores. GO and KEGG enrichment results demonstrated that the cellular functions in the preeclampsia group were focused on inflammation and immune responses, suggesting that the placental immune function of patients with preeclampsia was altered and different types of maternal cells interact and adapt to each other, therefore, rational immunomodulation is necessary for the normal development of the maternal placenta.

Hofbauer cells are placental macrophages within the placental chorionic mesenchyme, positioning in close proximity to the fetal vascular endothelium and cellular trophoblast, and are the only population of fetal-derived immune cells in the healthy placental mesenchyme that have been shown to have phenotypes associated with regulatory and anti-inflammatory functions [27, 28].They are present throughout gestationand normally exert immunosuppressive effects in normal pregnancy with M2 macrophage characteristics to maintain the immune tolerance [29]. Study has indicated that Hofbauer cells regulate pregnancy and placental morphogenesis through its involvement in vasculogenesis and angiogenesis [30]. They also regulate vascular endothelial cell growth and tissue remodeling by releasing high levels of VEGF and sprouty proteins. Ozmen et al. found that Hofbauer cells involved in the pathogenesis of preeclampsia by exerting a pro-inflammatory effect through the leptin-LEPR signaling pathway to promote endothelial cell dysfunction [31]. Our findings revealed the existence of three subtypes of macrophages, including macrophages and monocytes, as well as Hofbauer cells unique to the placenta, in which monocytes ultimately differentiated into macrophages and Hofbauer cells, which function as the disease progresses in preeclampsia.

The placental cells are required to expand aggressively to invade uterine tissues, remodel the maternal spiral arteries, form a network of blood vessels, and establish a maternal-fetal interface [11]. Therefore, the steady development of the placenta during the pre-gestational period is of paramount importance. When preeclampsia develops, it is likely to disrupt this homeostasis, leading to severe vascular defects in the embryo and placenta, and even causing early gestational fetal death [32, 33]. Our research showed that SPP1, Annexin, MIF, Visfatin, VEGF, Galectin, PTN, and CCL signalings were significantly inhibited among the three subtypes of macrophages in the preeclampsia group. Gabinskaya et al. reported that SPP1 belonged to the extracellular matrix proteins, and the absence of which causes failure of superficial cell trophectoderm invasion and insufficient arterial remodeling [34]. Deletion of related proteins in the Annexin family has been shown to result in aberrant metamorphosis and participation in the placental inflammatory response, impairing the uterine microenvironment that promotes embryo implantation and placenta formation [35, 36]. Furthermore, MIF signaling was associated with inflammation in the placental microenvironment [37]. Studies have been emphasized the potential future use of Vasfatin and Galectin as diagnostic markers for pregnancy complications [38, 39]. Pregnancy induces an increase in cardiac output, intravascular volume and systemic compliance [40], thus, VEGF is essential as a regulator of placental vascular endothelial growth and developmental homeostasis, and its absence leads to endothelial disorders. Taken together, these signaling changes in our findings were consistent with those that have been previously reported. These signals may therefore serve as potential signaling pathways in the development of preeclampsia.

Lastly, we found that the SLC9A9, SH2B3, SDC3, RCC2, F13A1, CCL2, and CBLB in macrophages were likely to be correlated with preeclampsia. Among these genes, abnormal CCL2 level was associated with preeclampsia [41, 42]. Study has reported that patients with preeclampsia presented lower serum levels of CCL2 [43]. Meanwhile, proteomic analysis by Yu et al. demonstrated that F13A1 was significantly down-regulated in the preeclampsia group compared to control, which was considered as a biomarker for preeclampsia diagnosis [44]. These were consistent with the trend of differential expressions of CCL2 and F13A1 in the transcriptome in our study. In addition, Zeng et al. proposed the potential involvement of SH2B3 in the occurrence of preeclampsia or preeclampsia with fetal growth restriction [45]. The potential role of other genes in preeclampsia remains to be further studied and discovered. However, this study has some limitations. First, the single-cell samples were small and our findings should be validated in large-scale studies containing datasets with larger sample sizes. In addition, the genes or signaling pathways associated with preeclampsia found in the study need to be further analyzed in conjunction with clinical samples. Currently, the complex regulatory mechanisms that exist between the placenta and the mother have not been fully clarified, so more in-depth and systematic studies are needed.

## Conclusion

This study analyzed the immune microenvironment in preeclamptic placental samples using single-cell transcriptome combined with RNA-sequencing, and found the important role of macrophages in regulating preeclampsia and obtained potential disease markers, which will provide new insights into the explanation of preeclampsia’s pathogenesis and new strategies for the clinical treatment of preeclampsia.

## Data Availability

The data that support the findings of this study are available from the corresponding author upon reasonable request.
